# Female Community Health Workers and Health System Navigation in a Conflict Zone: The Case of Afghanistan

**DOI:** 10.3389/fpubh.2021.704811

**Published:** 2021-08-11

**Authors:** Ateeb Ahmad Parray, Sambit Dash, Md. Imtiaz Khalil Ullah, Zuhrat Mahfuza Inam, Sophia Kaufman

**Affiliations:** ^1^BRAC James P Grant School of Public Health, The Center of Excellence for Gender, Sexual and Reproductive Health and Rights, BRAC University, Dhaka, Bangladesh; ^2^Melaka Manipal Medical College, Department of Biochemistry, Manipal Academy of Higher Education, Manipal, India; ^3^Advanced Intelligent Multidisciplinary Systems Lab, Department of Commuter Science Engineering, United International University, Dhaka, Bangladesh; ^4^University of Cambridge, Cambridge, United Kingdom

**Keywords:** community health workers, humanitarian crises, Afghanistan, humanitarian health, health workforce, medical assistance, accreditation, women working

## Abstract

Afghanistan ranked 171st among 188 countries in the Gender Inequality Index of 2011 and has only 16% of its women participating in the labor force. The country has been mired in violence for decades which has resulted in the destruction of the social infrastructure including the health sector. Recently, Afghanistan has deployed community health workers (CHW) who make up majority of the health workforce in the remote areas of this country. This paper aims to bring the plight of the CHWs to the forefront of discussion and shed light on the challenges they face as they attempt to bring basic healthcare to people living in a conflict zone. The paper discusses the motivations of Afghani women to become CHWs, their status in the community and within the health system, the threatening situations under which they operate, and the challenges they face as working women in a deeply patriarchal society within a conflict zone. The paper argues that female CHWs should be provided proper accreditation for their work, should be allowed and encouraged to progress in their careers, and should be instilled at the heart of healthcare program planning because they have the field experience to make the most effective and community oriented programmatic decisions.

## Introduction

Afghanistan, with one of the lowest Human Development Indexes in the world, ranked 171 among 188 countries in gender inequality index in 2011 with 16% of women labor force participation (1).

The country has been mired in violence for decades which has resulted in the destruction of social infrastructure, including the health sector. Following the involvement of US-led NATO forces in 2003, the new government established a Basic Package of Health Services (BPHS) with the help of international donors to deliver services to the majority of the rural population, with a particular emphasis on women and children (2).

Community health workers (CHWs) have recently been deployed in Afghanistan as part of the country's national health-care system and make up the majority of the health workforce in the remote areas of this country (3). CHWs are mostly community volunteers who are nominated by a Village Health Council (VHC) or Health Shuara—a community-based advisory committee charged with ensuring the smooth functioning of a health facility in a given region.

The basic package of health services for Afghanistan (BPHS) initiative does not require the CHWs to have any formal education (4). Instead, they are provided with highly targeted trainings to manage basic illnesses. These multi-phase trainings are conducted by the BPHS implementing NGOs, who also supervise them, using discussion, demonstration and storytelling methodologies for a period of minimum 8 weeks (5). Despite having no formal training and being non-health professionals, the CHWs of Afghanistan are often the first point-of-contact for most health-related matters. Table 1 shows the selection criteria, roles, responsibilities and compensation of the CHWs of Afghanistan in-detail. Female mobility in Afghanistan is largely limited due to cultural and religious norms. Thus, female CHWs are often accompanied by a Mahram—usually their husband, brother, or father who acts as a male religious guardian. The BPHS initiative is intended to employ such CHW couples/partner groups in order to ensure that health services are delivered efficiently and that neighborhood navigation is as simple as possible (6).

**Table 1:** Selection criteria, roles, responsibilities and compensation of CHWs in Afghanistan [Excerpt from Basic Packages of Health Services, Ministry of the Public Health (4)].

**The Community Health Worker**
A health post should have one male and one female CHW if possible. The MOPH encourages the training of couples assigned to the same health post. At least 40% of CHWs should be female. A health post serves a population of between 1,000 and 1,500 people, but in sparsely populated areas the population served may be as small as 400. Health program managers should give special attention to the coverage of communities by CHWs and train more if required to give access to the whole population.
** a) Changes to the job description in this revision of the BPHS**
∙ No major changes are introduced to the job description of the CHW in this revision. Some changes are introduced to the provision of birth spacing services and the management of diarrheal diseases. These are being supported by in-service training programs and changes to the preservice training. ∙ After appropriate training, CHWs will be allowed to counsel women on the use of DMPA and give the first injection as well as follow-up injections ∙ CHWs are encouraged to promote the Lactation Amenorrhea Method (LAM) of birth spacing in the first 6 months after a child is born, and then counsel women on the transition to another appropriate birth spacing method. ∙ Zinc therapy is introduced into the management of all diarrheal diseases. ∙ Cases of dysentery will need to be referred for treatment with Ciprofloxacin. ∙ CHWs will also be taught to be more aware of mental health and disability problems that can be helped, and how to refer such people.
** b) Job Description for the Community Health Worker (CHW)**
∙*Revised by the MOPH Community-Based Health Care Task Force, March 2005* ∙ The community health worker (CHW) is a person (female or male) selected by the community according to selection criteria reflected in the Policy on Community Health Workers (June 2003). The CHW promotes healthy lifestyles in the community, encourages appropriate use of health services, and treats and refers common illnesses. ∙ The CHW is accountable to the local Shura for performance and community satisfaction and technically accountable to the community health supervisor (CHS) assigned by authorities from the nearest health facility.
**General Responsibilities**
** A. Community Collaboration and Health Promotion**
1. Actively participate in community meetings and major community events. 2. Actively work with mother's groups to promote healthier homes and maternal and child health. 3. Encourage and mobilize family/community participation in the immunization of children and women of child-bearing age. 4. Support national initiatives at the village level and actively participate in all campaigns/activities e.g., National Immunization Days and surveillance for acute flaccid paralysis). 5. Promote good nutrition practices and encourage early breastfeeding and exclusive breastfeeding of children under 6 months of age for till the age of two. 6. Promote use of Oral Rehydration Salt (ORS) and other homemade rehydration fluids for home management of diarrhea and dehydration. 7. Promote hygiene and sanitation, and the preparation and use of safe drinking water. 8. Encourage couples to practice birth-spacing and receive family planning services. 9. Promote psychosocial well-being and mental health in the community and raise awareness about prevention identification of disability. 10. Create awareness within the community and provide information on the dangers of addictive substances such as tobacco, naswar, opium, hashish, and alcohol.
** B. Direct Services**
1. Identify and manage acute respiratory infections, diarrhea, malaria, and other common communicable diseases according to national protocols. Treat mild to moderate cases and refer complicated cases to the nearest health center. 2. Counsel patients on correct use of medications included in the CHW kit. 3. The CHW should create awareness among the community on how to prevent TB and should refer or accompany suspected cases to a health facility. Following completion by a tuberculosis patient of the first phase of treatment at the health facility, the CHW should ensure compliance of TB patients with the second phase treatment course in the community, based on DOTS. 4. Communicate the importance of antenatal and postnatal care. Distribute micronutrients and antimalarials to pregnant women according to national policy. Encourage the community to make regular and timely use of Maternal Child Health (MCH) services. 5. Encourage the use of skilled birth attendants, where possible, and help families to make birth plans. Provide and teach the use of a mini delivery kit. Teach family members to recognize the danger signs of complications of pregnancy and childbirth, and assist them in making preparations for emergency referral. 6. Distribute oral contraceptives and condoms to willing members of the target population according to national policy. Promote LAM together with exclusive breastfeeding for the child's health during the first 6 months of a child's life. Administer first and follow-up injections of Depo Provera. Encourage interested families to seek long-term family planning methods at a health facility. 7. Provide first-aid services for common accidents at the family and community level. 8. Ensure administration of vitamin A to children aged 6 months to 5 years during NIDs.
** C. Management**
1. Meet regularly with the Shura to develop, implement, and monitor community action plans for health improvement. 2. Meet regularly with the community health supervisor to review reports and action plans, receive supplies, and for in-service training. 3. Collaborate with and support community midwife activities in the catchment area, including health promotion and pregnancy-related referrals. 4. Regularly complete and submit the monthly Tally Sheets to the CHS for the HMIS. 5. Know the members of the community, and develop a community map of the eligible families in the catchment area and the services they have used. 6. Report all deaths and other activities included in the report form of the health post. Inform the health facility of any disease outbreaks. 7. Manage the health post, maintaining supplies and drugs given to CHWs and reporting utilization of drugs and supplies. 8. The establishment of Shurai Sehi at the HP level.
** D. Compensation for CHWs**
∙ CHWs should be compensated for all legitimate expenses (transport and food) when working outside their community. Specifically, approved under this BPHS revision: ∙ Afs100 per month for routine work travel ∙ Additional expenses (Afs50) for approved tasks like accompanying a suspected TB patient to a facility with a laboratory. On salaries, the MOPH 2005 policy continues: “The MOPH will not make regular payments to Community Health Workers (CHWs) from the MOPH budget and do not recommend donors' resources be allocated for regular payment to CHWs because such a policy is financially unsustainable.”
Communities are encouraged to support and compensate CHWs in traditional ways.

## Methodology

This paper uses a case study approach to understand what inspires women to work as CHWs and the difficulties they encounter as they work in the conflict-ridden rural areas of Afghanistan. It enlists several recommendations for the policy makers to integrate the CHWs in the formal healthcare system. This paper is a part of the larger research that was conducted as a part of the evaluation of the performance of one BPHS implementing NGO. This paper builds on the summary of the interviews and informal discussions commissioned by the Research and Evaluation division of BRAC Afghanistan for ethical approval and conducted with CHWs at Lashkargah city, in Helmand province of Afghanistan in September, 2019. A local NGO worker helped obtain verbal consent from the respondents as well as helping with translations and note taking during the interviews. The interviews were audio recorded and transcribed into the English. The data were consolidated into a data display matrix and thematic analysis was conducted accordingly following a pre-set framework that had the following themes; Motivation, Acceptance within the community, Economic exploitation of the CHWs, and Challenges faced while working in the conflict zones. Pseudonyms have been used to protect the privacy of the respondents. A sub-sample of the larger primary research, which includes first-hand accounts and observations along with the in-detail analysis of the interviews mentioned above mentioned above, has been analyzed into this perspective piece. Along with the primary data, this perspective also presents a triangulation of the opinions of the authors corroborated with secondary data.

## Motivation to Work as a CHW

Saira, a middle-aged CHW known as *Khala Saira* in the community and a mother of three children, had to bear the death of her first-born child to diarrhea. This tragedy particularly motivated her to work in community health so she could prevent another mother from the same trauma. Her main motivation as CHW was a variance of “altruism” and “monetary benefit.” She mentioned:

“*I chose to become [a CHW] because it helps me in [providing] services to my community. [Perhaps] I will get rewarded later for this work*”

The female CHWs mentioned that by obtaining knowledge, serving their communities, acting as a source of health advice and earning respect in their communities provided them with a sense of empowerment. Moreover, through this community work, many women also get mobility and the opportunity to socialize with other women, which they may otherwise lack. Suraiya, a young, married woman in her twenties also known in the community as *Suraiya Jaan* (beloved) benefitted from this. During training sessions, she was often found giggling and confidently interacting with other CHWs. Suraiya narrated that her husband was a “*Mulla*”—a religious leader, as was her father. These two men did not allow her to enroll in school or go outside of the house. She was married off when in her twenties and her husband happened to be a CHW. This was how her journey as a CHW started. In her words:

“*Becoming a CHW is [the only] blessing of my marriage. Now, I [can] go outside, meet my colleagues and encourage others to become CHW.”*

## Accepted by the Community, Yet Neglected by the Hierarchy

Although more than half the health workforce is women, they largely remain in low-paying positions including CHWs, nursing and midwifery. However, men continue to remain in high-paying managerial and policy-oriented health-related positions.

The limited social, financial, and political mobility of women in Afghanistan is exacerbated by the fact that female education is often restricted or limited; this contributes to their inability to obtain higher level positions in society that would afford them more stability, independence, and agency over decision-making.

When Fatima, another middle aged CHW, was asked why she still worked as a CHW even after 14 years of service, she replied:

“*I tried to learn to become a supervisor but the [health] manager did not allow me to, [rather] he sent younger women [for training] who were more educated and beautiful.”*

Due to the conservative nature of Afghan society and limited job opportunities, women face challenges in attaining education and getting promoted that males do not. When asked why she did not try for any other supervisory position with her robust experience, she smiled and said:

“*I am a woman. How can I become a manager? It is for men […] I am more [comfortable] in the community where my people need me.”*

With this sentiment, she attributed her inability to be promoted to gendered power relations, norms and stereotypes. Other CHWs also believed that women are seen to be incapable of managerial roles. Female CHWs have immense demand and acceptance in the community, especially in contexts where women seek women health care providers. Due to this demand, paired with the existing societal limitations and gender-based division of labor, women working as a part of the healthcare workforce are compelled to be midwives, nurses, CHWs and in many cases, cleaners with no career development opportunities.

Nevertheless, their acceptability in the community is extremely high and they are perceived as and called “Village Doctors” by those in their communities. When Khala Zarina, another middle-aged CHW, was asked about the biggest strength the CHWs possess, she mentioned “*being able to provide Maternal and Child health (MNCH) services*.” Khala Zarina mentioned that male CHWs are not well-accepted by the community for MNCH services, and that the heads of the family (often male) never consider their advice regarding pregnant mothers. Describing a scenario, she said,

“*Many [pregnant] women face difficulties in delivery. [In such cases] Male CHWs cannot be called [due to religious barriers]. But since we [female CHWs] took over, people immediately call CHWs even if they live far away…”*

This is a prime example of how female CHWs are perceived to be and act as the best bridge to link communities with community-centric health systems. Yet, their role, effort and contribution in the broader health system is ignored. Rather, they are considered auxiliaries of weak health systems.

## Economic Exploitation

CHWs in Afghanistan are basically Community health Volunteers (CHVs) who are not salaried. Instead, they are reimbursed for their travel expenses amounting to $2USD per month. The lack of compensation creates serious hardships for many CHWs, especially those who are women. All of the CHWs who were interviewed lived in economically depressed areas, with some living in areas absent of any health facility. This led them to be the sole providers of any health services.

Khala Saira, Suraiya and Fatima mentioned that often their reimbursements are delayed, being paid 4–8 months late. Some of the CHWs relied on this reimbursement and monthly incentive to support their families during desperate times. For Suraiya, the situation was slightly different as her husband, also a CHW, would always receive the money on her behalf,

“*It does not matter to me when they pay [the money]. I don't get to see it [anyways]. My husband receives it.”*

Similarly, other CHWs' payments were also controlled by the male heads of their families. This is a common scenario in Afghanistan. For those who are married, husbands control the income and for unmarried women, fathers take the earnings. In such cases, the delayed payment can increase the threat of physical and psychological violence toward these women. For the CHWs who are the main financial contributors in their families, as was the case of Khala Saira, the 4–8-month delayed payment can be catastrophic, creating debts and instability.

## Threats and Violence Against CHWs

The CHWs usually provide services in health posts. However, in some cases such as midwifery and birth delivery, the CHWs visit the patient's house to deliver the needed health services. For some female CHWs, venturing outside of their homes is challenging. Additionally, domestic violence can be an issue as these female CHWs have to navigate a patriarchal society like Afghanistan.

Community members often call CHWs late at night for help. This is frowned upon by their families and often poses further challenges. For instance, Khala Zarina mentioned that she once received a call late at night from a man whose wife was bleeding from a recent delivery. However, Zarina's husband was so offended at this that he started beating her in front of the other man:

“*My husband got so angry when someone came [knocking] at our door. He needed help for his wife. But my husband abused him and started beating me. My hand fractured and [I] could not move it for a month.”*

## Harassment

Many female CHWs face harassment from their male colleagues and supervisors and have to deal with these issues by themselves as social stigma prevents them from lodging complaints. Talvasa, an unmarried CHW who worked in pair with her physically challenged brother, narrated how her male supervisor attempted to take advantage of her. Talvasa kept the concern to herself because in such cases the society, controlled by men, blames the women. She said:

“*My supervisor was not happy with my work. I tried my best, but he always complained and threatened me. One day [he] asked me to meet him in a health post after 3 PM. I ran away from the office and did not meet him for many days.”*

Similarly, a male cashier threatened Khala Saira. He didn't pay her payment/incentive for months and then 1 day asked her to meet him after office hours when he said he would pay her. However, Khala Saira denied this invitation. In her own words,

“*When one cashier asked me [to] spend time with him after office [hours]. I told him that dying is better than killing my conscience.”*

Although somewhat covert, both Khala Saira and Talvasa are hinting at the rampant sexual harassment and exploitation that exists when men in power withhold a deserved and earned payment or position unless provided something additional, often sexual, in exchange.

Sometimes, even the community members harass, humiliate and abuse the CHWs, particularly during mass vaccination drives. Afghanis often stigmatize vaccinations because they see it as a western propaganda. Hence, when a CHW attempts to provide vaccination or vaccination-related information to community members, they often face abuse from the men. In some cases, sexual harassment also becomes rampant when they enter the homes to vaccinate. Talvasa narrated that once while providing a tetanus-toxoid immunization to a pregnant woman, the man in the house forcibly held her hand and pushed her against the wall. He then whispered in her ear,

“*You have no shame. Why do you come again and again, door to door? Is your husband incapable?”*

She mentioned that his wife lay on the floor and could not say anything as she watched the whole scene. Talvasa cried while she said how many similar incidents had traumatized her but she was unable to share her feelings with her family out of shame and fear.

## Stuck Between Health Posts and Battle Zones

Orthodox Fundamentalists (AGEs) in Afghanistan pose a serious threat for the CHWs. AGEs oppose female employment, immunization campaigns, and family planning. As such, the AGEs have restricted the entry of health workers or the establishment of health facilities in many areas they control. As a result, the community is suffering from many preventable conditions like Polio (7).

Khala Zarina mentioned that she was threatened by armed men while she was counseling a group of women on child vaccination. However, this type of threat and harassment does not stop CHWs from doing their jobs. Commitment to the work and the ideals of health for all, perseveres and is promoted by these heroic women every day. In the words of Yasmin, a middle-aged CHW,

“*I fear going to the Health post because [often] gunmen come and take away all the medicine to battle zones. They also stare at us and threaten to kill our husbands and take us with them. But this is my work. If not me, who else will do it?”*

## Discussion

This article sheds light on the plight and limitations of Afghan female CHWs and also highlights the specific health obstacles that exist for those giving and receiving care in conflict zones.

Afghanistan's history of internal conflicts and war, instability, and on-going conflict has deeply impacted the region and the health of the country. The conflict has not only destroyed and depleted health centers and resources, it has also created refugee populations in areas which lack adequate health services. It has also negatively impacted immunization and led to landmines and related accidents of the country. It has also led to a decrease in the health outcomes particularly linked with maternal health (8). While healthcare has been implemented in the country through various humanitarian aid programs, they have primarily been provided by foreigners which has left the healthcare system dependent on outside actors. This history calls for localization of healthcare workers.

Gender-related risks and obstacles have been historically detailed in countries of conflict including Afghanistan, including but not limited to increased gender-based violence. In Afghanistan, conflict calls women to perform new duties and fill new roles. From the research above, it becomes apparent that these new roles can be either empowering or disempowering depending on how they are harnessed and supported.

Female mobility, independence, social roles, access to education and work opportunities have been directly impacted by country conflict. One study conducted by Physicians for Human Rights reported that high maternal mortality and limited female mobility in Afghanistan was directly correlated with the depletion of women's rights and freedoms that occurred due to the conflict and the Taliban regime (9). Working individually or as a local “unit” without the support of large outside or foreign agencies such as the UN, the female CHWs are in a unique situation that calls for increased protection mechanisms and mobility.

This article encompasses the numerous socio-cultural and political obstacles that hinder the smooth functioning of CHWs. First, their irregular status and lack of financial assistance from the BPHS program poses a huge obstacle. The experiences of CHWs, who are predominantly found in low and middle income countries (LMICs) (10), needs to be seen both from an “altruistic capital” point of view and the monetary compensation angle. Altruistic capital refers to the intrinsic motivation that the CHWs have (11). It is seen from the narratives in this paper that intrinsic motivation is paramount within Afghan women CHWs. However, inadequate financial compensation, delayed payments and unrealistic expectation of such part time models can soon deplete the reservoir of intrinsic motivation (12). Second, CHWs are refused the ability to progress in their careers. The troublesome truth of “once a CHW, always a CHW” is vividly illustrated by Khala Fatima's narrative, where working as a CHW is the only choice for women working in health sector. In a study with CHWs in India, one of the major factors that affect performance of CHWs was shown to be lack of job security and career progression. A lack of clear affiliation, lack of reward for good performance, lack of attributing “employee status” are factors that demotivate CHWs (13, 14). We believe that the status of CHWs should be supported and acknowledged within Afghanistan's formal healthcare framework. Furthermore, they should be given training and encouraged to advance to supervisory roles where they can apply their knowledge and contribute to program planning, design, and execution.

Afghanistan could adopt newer or alternate models of working with CHWs. For example, in sensitive conflict zones in Burma, instead of clinic workers as CHW, local residents like teachers and retired health workers were recruited (15). In neighboring Pakistan, in a post conflict period, a technology-based psychological treatment program, Thinking Healthy Program (THP), was successfully delivered by CHW to battle perinatal depression (16). Such a “quick win” model could be adopted in Afghanistan as it has a similar societal structure and the model would help remove the important barrier of “acceptance” of CHWs and their work.

Finally, many of the CHWs' abuse reports illustrate how exploitation and gender discrimination jeopardize the country's overall public health programs, which are a problem for everyone (17). National studies have shown that 52% of women reported having faced domestic violence. Wife beating is normalized, riding on deep conditioning and lack of awareness among women (18). These social determinants of health need to be factored when roles and responsibilities of CHWs are evaluated. To avoid such incidents and ensure that no CHWs are left vulnerable, public health agencies must specifically identify safeguarding policies. The CHW's position in fostering health in Afghanistan is crucial, and it is past time for them to be recognized as such.

Encouraging and supporting the work of female CHWs that provide opportunities for upward mobility are necessary for sustainable health systems strengthening in conflict prone zones (19) including Afghanistan. Moreover, encouraging their career advancement and protection measures against abuse are critical in humanitarian conflict zones. This research illuminates the complex relationship that can exist between healthcare delivery, social and cultural belief systems, and mercurial regional politics in Afghanistan, poised for yet another change in the course of the nation state with the Biden administration implementing its policies (20). It also emphasizes how healthcare interventions must incorporate each of these elements for systems improvement.

## Data Availability Statement

The datasets generated for this article are not readily available because Due to the sensitive nature of the data and the challenges mentioned by the CHWs pertaining to their work in the AGE-controlled zones of Afghanistan, the ethics committee did not approve public sharing of the datasets used in this study. However, the working dataset used for this paper is available on request. Requests to access the datasets should be directed to ahmad.ateeb101@gmail.com.

## Ethics Statement

The studies involving human participants were reviewed and approved by Research and Evaluation Division, BRAC Afghanistan. Written informed consent for participation was not taken for this study due to the preference of respondents.

## Author Contributions

AP initiated a draft of key conceptual points to which all authors inputted and coordinated the inputs and edited the final manuscript. All authors made text input to iterative drafts and provided reference materials using shared online document editing software.

## Conflict of Interest

The authors declare that the research was conducted in the absence of any commercial or financial relationships that could be construed as a potential conflict of interest.

## Publisher's Note

All claims expressed in this article are solely those of the authors and do not necessarily represent those of their affiliated organizations, or those of the publisher, the editors and the reviewers. Any product that may be evaluated in this article, or claim that may be made by its manufacturer, is not guaranteed or endorsed by the publisher.
